# Sleep Quality as a Mediator in the Relationship Between Perceived Stress and Job Burnout Among Chinese Nurses: A Structural Equation Modeling Analysis

**DOI:** 10.3389/fpsyt.2020.566196

**Published:** 2020-11-13

**Authors:** Yang Song, Fengzhi Yang, Kristin Sznajder, Xiaoshi Yang

**Affiliations:** ^1^School of Fundamental Sciences, China Medical University, Shenyang, China; ^2^School of Public Health, China Medical University, Shenyang, China; ^3^College of Medicine, Pennsylvania State University, Hershey, PA, United States

**Keywords:** burnout, perceived stress, sleep quality, nurses, structural equation modeling

## Abstract

**Background:** Job burnout has become an increasing prevailing phenomenon among nurses in both developed and developing countries. There is a paucity of research exploring the relationship between perceived stress (i.e., the level of one's perception or appraisal of stress rather than objective stressful events) and job burnout and no existing literature examining the mediating role of sleep quality in the relationship between these two constructs. The objective of the study was to examine if sleep quality mediates the relationship between perceived stress and job burnout.

**Methods:** Cross-sectional data were collected from a total of 1,013 nurses working in six public tertiary hospitals in China. The self-administered questionnaire included demographic information, the Maslach Burnout Inventory-General Scale, the Pittsburgh Sleep Quality Index and the Perceived Stress Scale. Hierarchical multiple regression (HMR) analyses were performed to examine the contribution of each covariate to the prediction of job burnout. Structural equation modeling (SEM) was employed to test whether the proposed relationships between variables involved existed.

**Results:** Both perceived stress and poor sleep quality exhibited strong positive associations with job burnout among Chinese nurses. The SEM analysis confirmed the direct pathway from perceived stress to burnout and the indirect pathway mediated by sleep quality. The direct effect of perceived stress on job burnout was found to be statistically significant and positive (β = 0.69, *p* < 0.05). There existed statistically significant effects of sleep quality on both perceived stress (β = 0.48) and job burnout (β = 0.29). The path coefficients of perceived stress on job burnout were significantly reduced (β = 0.56) when sleep quality was modeled as a mediator. The bias-corrected and accelerated bootstrap test revealed that sleep quality had a significant mediating effect on the relationship between perceived stress and job burnout (a ^*^ b = 0.139, BCa 95%, CI: 0.110~0.174).

**Conclusion:** Perceived stress might exert significant effects on burnout both directly and indirectly through the mediating role of sleep quality. Efforts to reduce burnout among nurses in clinical settings may benefit from interventions for coping with perceived stress and practices for promoting healthy sleep.

## Introduction

Job burnout has been recognized as an occupational health problem that occurs during long-term exposure to work-related stress and may involve multiple symptoms (1). Emotional exhaustion, depersonalization, and diminished personal accomplishment are three specific characteristics constituting burnout (2) and World Health Organization has recently classified it as a syndrome “resulting from chronic workplace stress that has not been successfully managed” (3).

Nurses have long been considered as working in a profession where they are confronted with a wide range of stressors including emotionally demanding patient contacts, workload and time pressure (4), ever-changing technology and institutional and ethical challenges (5), and often being confronted with insufficient resources to effectively cope with high job demands (4). Long-term exposure to stress is common in nursing, which may affect up to 71% of nurses (6) and cause mental, physical and emotional exhaustion and subsequently result in burnout (7).

Burnout, though depending on individual peculiarities, can be linked to a wide variety of detrimental personal and organizational outcomes. Nurses have been found to be particularly susceptible to burnout in hospital settings (8). Burnout can manifest in both physical and psychological symptoms including, among others, weakness, insomnia, anxiety, depression, hostility, aggressiveness (9, 10). Burnout can also lead to reduced work effectiveness and job satisfaction (11) and higher levels of absenteeism and turnover among nurses (10). Furthermore, it can result in decline in patient satisfaction and the quality of nursing care (12, 13). Although reports of the rates of burnout among nurses have varied, burnout has become an increasing prevailing phenomenon in different nursing specialties and among nurses in both developed and developing countries. It has been documented that the level of burnout has been increasing steadily over the recent years among Chinese nursing staff (14), affecting up to 68.1% in the existing literature (15).

Recent literature has found sleep quality had an association with the occurrence of burnout syndrome and contributed to nurses' recovery from fatigue and psychological stress caused by work (16). Poor sleep quality may not only result in health problems of the health care personnel but also may be linked to impaired clinical performance and higher risk of medical errors that may jeopardize the safety of patients (17). It has been widely recognized as a critical issue among the nursing staff. It was found that up to 55% of clinical nurses working in general hospitals in Mainland China were susceptible to sleep problems (18). A cross-sectional study conducted among Turkish nurses reported that 79.1% of the participants experienced poor sleep quality and those with poor sleep quality were found to experience more sleepiness at work and have higher levels of burnout (19). Adequate amount of sleep has been found to be a protective factor for burnout, which helps nurses restore energy and recover from the exhaustion of working days (20). In Medscape's newly-released 2020 National Report on physicians' burnout, millennial physicians are most likely to resort to sleep to cope with burnout (21). Longer sleep onset and more fragmented sleep has been associated with greater levels of burnout (22). Short sleep duration was shown to be common among the nursing staff. It has been documented that there was lower likelihood of job strain and burnout for those who slept 7 h or longer every working day (23) and fewer hours slept on average served as a predictor of higher depersonalization (24).

Although studies have yielded results that indicate the association of sleep quality with work-related stress and burnout independently among the nursing staff, there is no existing literature examining whether sleep quality plays a mediating role between stress and burnout. Also, despite that fact that stress has been documented as a predictor of burnout in numerous theoretical and empirical studies, stress propensity varies between individuals and the degree to which stress exerts a negative impact depends on one's perception or appraisal of stress (25). One's response to stressful events (often referred to as stressors) is determined by how he or she perceives or appraises stress rather than the objective occurrence of events (26). Research focusing on the effect of perceived stress (i.e., the level of appraised stress rather than objective stressful events) on burnout is scarce and such research has not yet been conducted among nurses in Chinese clinical settings. In this study, we hypothesized that perceived stress could have positive effect on job burnout and sleep quality could act as a mediator in the relationship between perceived stress and job burnout. Based on the research findings reported above, the objective of the study was to explore: (1) whether a relationship between perceived stress and burnout exists among Chinese nurses; (2) whether sleep quality could act as a mediator in the relationship between perceived stress and job burnout.

## Materials and Methods

### Research Design and Sample Selection

The current study was based on cross-sectional data with propositional sampling collected from October 2017 to February 2018 among 1,300 nurses working in six public tertiary hospitals in Shenyang the capital of Liaoning province, the largest city in northeastern China. Approximately twenty-five percentage of the nurses who work in the clinical departments including medical, surgical, gynecological, pediatric, and other divisions were chosen randomly from each of these six hospitals. Eligibility criteria for the participants include: (1) licensed as a registered nurse; (2) working in a nursing position for more than 1 year; (3) voluntarily participating in the present study. A self-administered questionnaire, which took approximately 25 min to complete, was distributed. A total of sample of 1,013 nurses provided effective responses to the questionnaire and constitute our final participants, which achieved an effective response rate of 77.92%.

### Ethics Statement

The study was conducted in accordance with the ethical guidelines and approved by the Committee on Human Experimentation of China Medical University. The study protocol was fully explained and written informed consent was obtained from each participating nurse before the initiation of study procedures. Nurses' participation in the study was entirely voluntary and anonymous; their personal information and responses were held in strict confidentiality.

### Instruments

Demographic characteristics examined in this study included age, gender, marital status, level of education, and monthly income. “Educational level” was categorized as “Junior college or below” and “bachelor or above.” “Marital status” was classified as “Married or cohabiting” and “other.” “Monthly income” was divided into “ <3,000 RMB” and “≥3,000 RMB.”

Job burnout was measured with the Maslach Burnout Inventory-General Scale, which is the most widely used measurement tool of burnout (27). It assesses burnout in three subscales; emotional exhaustion, depersonalization, and personal accomplishment. The Chinese version consists of 15 items including five items measuring emotional exhaustion, four items measuring the depersonalization and six items measuring personal accomplishment. Each item is scored on a seven-point frequency scale ranging from 0 (“never”) to 6 (“daily”), with higher scores in the subscales of emotional exhaustion and depersonalization and lower scores in the subscale of personal accomplishment indicative of higher levels of burnout. In the present study, the Cronbach's alpha for the scale was 0.899.

Sleep quality was assessed with Chinese version of the Pittsburgh Sleep Quality Index. The Pittsburgh Sleep Quality Index is the most commonly used instrument to assess quality of sleep in clinical and research settings (28, 29). It comprises 19 self-rated items measuring sleep quality in seven dimensions including subjective sleep quality, sleep latency, sleep duration, habitual sleep efficiency, sleep disturbances, use of sleeping medications and daytime dysfunction (28). Each item is scored on a Likert scale ranging from 0 to 3, indicative of the extent from “no difficulty” to “severe difficulty.” A higher global score represents poorer sleep quality. The Chinese version of Pittsburgh Sleep Quality Index has been validated as a highly reliable instrument for measuring the sleep quality among Chinese nurses (16). In the present study, the Cronbach's alpha for the scale was 0.697.

Perception of stress was measured by the Perceived Stress Scale (PSS), which was developed by Cohen (26) and has been extensively used as a classic stress assessment instrument for measuring the perception of stress. The Chinese version of the 10-item Perceived Stress Scale has been shown to have good reliability and validity (30). Items in this scale included “In the last month, how often have you been upset because of something that happened unexpectedly?” and “In the last month, how often have you felt difficulties were piling up so high that you could not overcome them?” each of which was reported on a 0–4 scale ranging from “never” to “very often,” with higher scores indicative of higher levels of perceived stress. In the present study, the Cronbach's alpha for the scale was 0.808.

### Statistical Analysis

The data were analyzed using SPSS version 17.0. The differences in job burnout among categorical groups were examined using *t*-tests and one-way ANOVA. The correlations between three dimensions of job burnout, perceived stress and sleep quality were explored using correlation analysis. Hierarchical multiple regression (HMR) analyses were performed to examine the contribution of each covariate to the prediction of job burnout. Job burnout was used as the dependent variable and the independent variables (i.e., demographic characteristics, perceived stress, and sleep quality) were entered at subsequent steps. The following criteria should be met based on the approach proposed by Baron and Kenny (31) for establishing mediation: (1) the independent variable (perceived stress) is a significant predictor of the dependent variable (job burnout); (2) the independent variable (perceived stress) is a significant predictor of the potential mediator (sleep quality); (3) the potential mediator (sleep quality) is a significant predictor of the dependent variable (job burnout); (4) the effect of the independent variable (perceived stress) on the dependent variable (job burnout) is significantly reduced or no longer statistically significant when the mediator (sleep quality) is introduced. The Sobel test using structural equation modeling (SEM) was conducted to confirm the mediating effect of sleep quality on the relationship between perceived stress and job burnout. The goodness-of-fit index —χ2/df < 5, GFI, CFI, TLI > 0.90, and RMSEA < 0.08 was considered to indicate an adequate model fit. It could be speculated that sleep quality might have a mediating effect if a decrease in the size of direct path coefficients of perceived stress on job burnout or a disappearance of statistical significance could be observed upon the inclusion of sleep quality as a potential mediator in the model. The bootstrapping strategy was performed to test the mediating effect (a^∧^b product) of sleep quality on the relationship between perceived stress and job burnout (32). The bootstrap estimates were calculated based on 5,000 resamples and 95% bias-corrected and accelerated confidence intervals. Significance of a mediating effect was determined if the value of zero was outside confidence interval. All statistical tests were two-tailed with *P*-values < 0.05 being considered statistically significant.

## Results

The demographic information of participants and the corresponding distribution of job burnout are shown in Table 1. Among the 1,013 nurses, 38 (3.8%) were male and 975 (96.2%) were females. Four hundred and nineteen (41.4%) fell within the <30 age group and (714) 70.5% had bachelor's or higher degrees. Significant differences were observed in scores of job burnout, in particular, the subscale of personal accomplishment, regarding age, gender, marital status, education level and monthly income. Specifically, significantly higher levels of personal accomplishment were also observed in those aged 30 or above, those who are currently not married or cohabiting, those who had bachelor's or higher degree, and those who earned no <3,000 RMB per month (*P* < 0.01).

**Table 1:** Demographic characteristics and the distributions of job burnout among nurses (*N* = 1,013).

**Variables**	***N* (%)**	**EE (Mean±SD)**	**DE (Mean±SD)**	**PA (Mean±SD)**
Age (yrs)				
<30	419 (41.4)	11.91 ± 6.68	10.17 ± 6.79	19.76 ± 8.71
≥30	594 (58.6)	12.28 ± 6.80	10.37 ± 7.12	21.86 ± 9.37**
Gender				
Male	38 (3.8)	10.47 ± 6.03	9.92 ± 6.23	17.03 ± 9.70
Female	975 (96.2)	12.19 ± 6.77	10.30 ± 7.01	21.15 ± 9.10**
Marital status				
Married or cohabiting	682 (67.3)	11.80 ± 6.95	10.22 ± 6.91	19.79 ± 8.71
Others	331 (32.7)	12.28 ± 6.65	10.32 ± 7.02	21.57 ± 9.32**
Education				
Junior college and below	299 (29.5)	11.68 ± 6.52	10.03 ± 6.71	19.08 ± 9.74
Bachelor degree and above	714 (70.5)	12.31 ± 6.84	10.40 ± 7.10	21.79 ± 8.78**
Monthly income				
<3,000	281 (27.7)	11.97 ± 6.90	10.60 ± 6.99	19.62 ± 8.88
≥3,000	732 (72.3)	12.19 ± 6.69	10.17 ± 6.98	21.52 ± 9.21**

* *Significant at the 0.05 level (two-tailed)*;

** *Significant at the 0.01 level (two-tailed). Values are presented as mean ± standard deviation. EE, Emotional exhaustion; DE, Depersonalization; PA, Personal accomplishment*.

The correlations between different dimensions of burnout, perceived stress and sleep quality are presented in Table 2. The results reveal that significant correlations exist between all three dimensions of burnout and perceived stress (*P* < 0.01) and sleep quality (*P* < 0.01). Specifically, perceived stress (PS) was positively correlated with emotional exhaustion (EE), depersonalization (DE), and personal accomplishment (PA). Poor sleep quality (SQ) was associated with increased levels of all three dimensions of burnout. As shown in Table 2, the effect-sizes of the correlations of Perceived stress with Emotional exhaustion and Depersonalization were above 0.3 respectively, which indicated a medium effect-size with both practical and statistical significance; the effect-size of the correlation between Perceived stress and Personal accomplishment was above 0.1, which was small being statistically significant but practically negligible. Also, the effect-sizes of the correlations of Sleep qualtiy with Emotional exhaustion and Depersonalization were above 0.3 respectively, which indicated a medium effect-size with both practical and statistical significance; the effect-size of the correlation between Sleep quality and Personal accomplishment was above 0.1, which was small being statistically significant but practically negligible. *R*^2^ and adjusted *R*^2^ were included in Table 3 to better reflect the size-effects of the correlations of Job burnout with Perceived stress and Sleep quality.

**Table 2:** The effect size of continuous variables.

	**1 EE**	**2 DE**	**3 PA**	**4 PS**	**5 SQ**
1 EE	1				
2 DE	0.760**	1			
3 PA	0.132**	0.015	1		
4 PS	0.484**	0.412**	0.271**	1	
5 SQ	0.524**	0.448**	0.088**	0.335**	1

* *Significant at the 0.05 level (two-tailed)*;

** *Significant at the 0.01 level (two-tailed). EE, Emotional exhaustion; DE, Depersonalization; PA, Personal accomplishment; PS, Perceived stress; SQ, Sleep quality*.

**Table 3:** The hierarchical linear regression analysis of job burnout.

	**Job burnout**			**EE**			**DE**			**PA**		
	**Model 1**	**Model 2**	**Model 3**	**Model 1**	**Model 2**	**Model 3**	**Model 1**	**Model 2**	**Model 3**	**Model 1**	**Model 2**	**Model 3**
Block 1 Demographic characteristics												
Age (<30 vs. ≥30)	−0.042	−0.049	−0.078*	−0.010	0.002	0.038	−0.019	−0.008	0.023	−0.068	−0.062	−0.064
Gender (male vs. female)	0.007	0.013	0.013	−0.044	−0.035	−0.036	−0.007	0.000	0.000	−0.073*	−0.068*	−0.068*
Marital status (Married or Cohabiting vs. others)	0.011	−0.019	−0.022	0.021	−0.022	−0.021	0.001	−0.036	−0.035	0.022	−0.001	−0.001
Education (Junior college and below vs. Bachelor degree and above)	−0.019	−0.034	−0.054	−0.032	−0.010	0.009	−0.026	−0.007	0.010	−0.102**	−0.091**	−0.091**
Monthly income												
(<3,000 *vs*. ≥3,000)	−0.034	−0.036	−0.039	−0.001	0.004	0.007	0.037	0.041	0.044	−0.052	−0.050	−0.050
Block 2 Perceived stress		0.328**	0.201**		0.485**	0.351**		0.416**	0.301**		0.256**	0.262**
Block 3 Sleep quality			0.391**			0.413**			0.356**			−0.019
*R*^2^	0.004	0.110	0.244	0.005	0.236	0.385	0.002	0.173	0.284	0.035	0.099	0.099
Adjusted *R*^2^	−0.001	0.105	0.239	0.000	0.231	0.381	−0.003	0.168	0.279	0.033	0.097	0.096
*ΔR*^2^	0.004	0.106	0.134	0.005	0.231	0.150	0.002	0.171	0.111	0.035	0.064	0.000

* *Significant at the 0.05 level (two-tailed)*;

** *Significant at the 0.01 level (two-tailed). EE, Emotional exhaustion; DE, Depersonalization; PA, Personal accomplishment; PS, Perceived stress; SQ, Sleep quality*.

As is indicated in Table 3, perceived stress was significantly positively correlated with emotional exhaustion, accounting for 23.1% of the variance. Poor sleep quality was also significantly positively correlated with emotional exhaustion, explaining an additional 15% of the variance. The results indicate the potential effect of perceived stress on emotional exhaustion among Chinese nurses might be partially mediated by sleep quality. The regression coefficient (β) for the association between perceived stress and emotional stress was reduced from 0.485 to 0.351 when sleep quality was added to the model.

**Figure 1:**
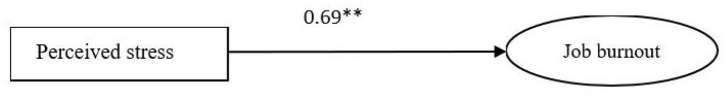
Structural equation modeling of perceived stress and job burnout.

**Figure 2:**
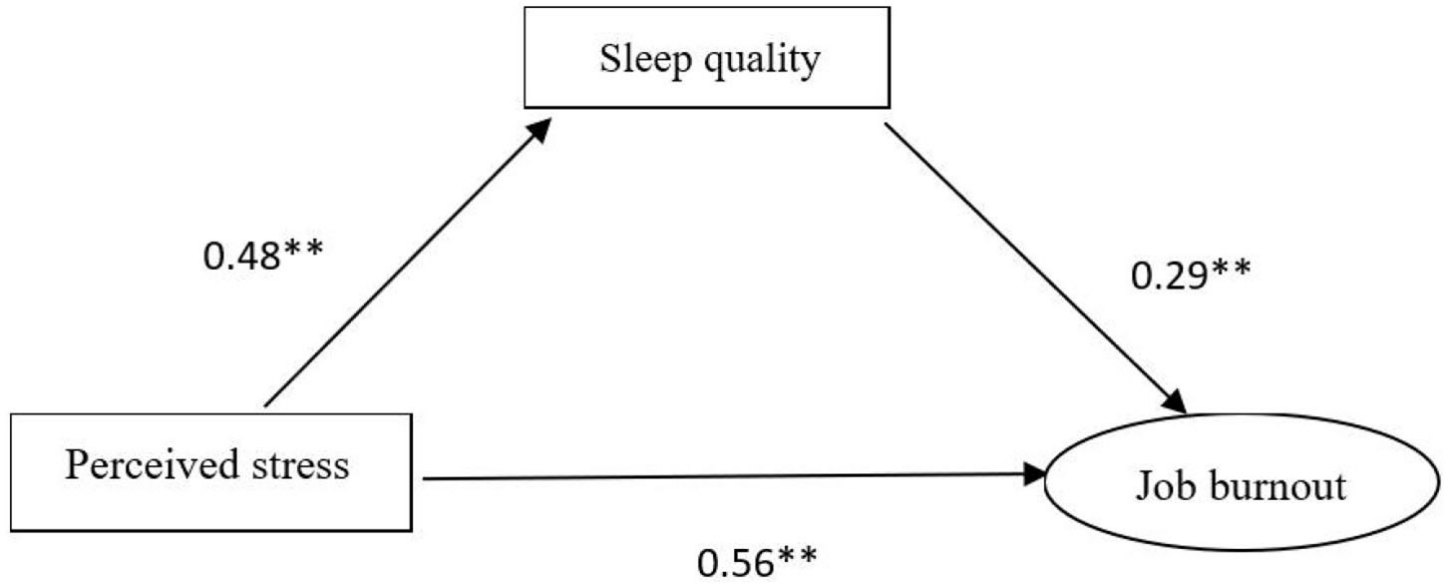
Structural equation modeling of the mediating role of sleep quality on the relationship between perceived stress and job burnout.

The statistical significance of the mediating effect was further confirmed by the Sobel test. The SEM yielded a good fit to the observed data indicating the direct pathway from perceived stress to job burnout and the indirect pathway which was mediated by sleep quality. As is shown in Figure 1, the direct effect of perceived stress on job burnout was estimated in the model (the model fit of the data χ 2/df < 5, *p* < 0.05, GFI = 0.970, AGFI = 0.939, CFI = 0.976, TLI = 0.958, and RMSEA = 0.060), which was found to be statistically significant and positive (β = 0.69). As illustrated in Figure 2 (the model fit of the data χ 2/df < 5, *p* < 0.05, GFI = 0.960, AGFI = 0.930, CFI = 0.968, TLI = 0.952, and RMSEA = 0.061), there existed statistically significant effects of sleep quality on both perceived stress (β = 0.48) and job burnout (β = 0.29). The path coefficients of perceived stress on job burnout were significantly reduced (β = 0.56) when sleep quality was modeled as a mediator. The bias-corrected and accelerated bootstrap test revealed that sleep quality had a significant mediating effect on the relationship between perceived stress and job burnout (a ^*^ b = 0.139, BCa 95%, CI: 0.110~0.174). Thus, it was confirmed that perceived stress might not only directly affect job burnout but could also exert a significant indirect effect on job burnout via sleep quality.

## Discussion

Based on our knowledge, this study was the first to explore the relationship between perceived stress, sleep quality and job burnout among Chinese nurses and also the first to examine sleep quality as a mediator in the relationship between perceived stress and job burnout. Results of this study illustrated that 66.34% of Chinese nurses experienced burnout, which is similar to the finding of another cross-sectional study conducted among nurses working in municipal hospitals in China reporting that 68.1% of Chinese nurses suffered from burnout (15). One study involving 10,319 medical-surgical nurses of 303 hospitals in US, UK, Canada and Germany found an incidence of burnout ranging from 32% in Scotland to 54% in US (33). Also, it was reported that 20–40% of Australian nurses had burnout symptoms (34, 35), while findings from another cross-sectional study showed that Australian nurses experienced higher burnout than their Chinese counterparts (36). Burnout among nurses has become a prevalent problem which needs to be taken seriously and addressed in both developed and developing countries.

Our study revealed that perceived stress has exhibited a strong positive relationship with burnout among Chinese nurses. The association between burnout and stress could be bidirectional, though burnout has typically been considered as a response to job-related stress (37, 38). It has been suggested that the occurrence of burnout might convert a previously enjoyable activity into a repelling source of stress (37). Victims of burnout are susceptible to low energy and chronic fatigue, which might lead to exhaustion during the day and poor sleep at night. From the emotional perspective, those with burnout tend to experience emotional depletion and difficulty feeling positive emotions. Feeling emotionally depleted may also give rise to tension and generalize negative attitudes to other aspects of life (37). From the physiological level, one's appraisals play an important role eliciting their physiological responses, which may also affect appraisal and reappraisal as part of feedback loop. That is, bodily responses of fatigue contribute to the generation and reinforcement of appraisals of overload (37). This finding lends support to existing studies conducted among dental students (39), athletes (40), and teachers (41) in different countries indicating that cognitive appraisals of stressors could play a part in determining one's susceptibility to burnout. Previous studies have suggested that stress in and of itself is neutral (42) and it is the perception or appraisal of stress that plays a determining role in whether stressors have a positive or negative impact (25). One's response to stressors is determined by his perception of the stressfulness instead of the objective stressful events (26). Whether an event or a situation is appraised as threatening or challenging depends on one's availability of coping resources or if there exists an imbalance between demands and resources for a prolonged period of time (25).

Despite the fact that China's healthcare system is undergoing rapid development, there still exist a multitude of challenges that remain to be addressed. The percentage of the population aged 60 and over in China exceeded 12% in 2012 and an increasing trend of age-standardized mortality rates due to non-communicable diseases were observed (43), which put enormous strain on China's nursing system (44). Moreover, due to rapid socioeconomic growth and increased lifespan, there is an increasing demand for better-quality health care in China, with patients seeking more specific and personalized health-care services (45), which results in an increase in the workload of the nursing staff to deliver high-quality nursing care to meet the patients' needs (46). On the other hand, although the density of registered nurses per 1,000 population in China increased dramatically from 1.52 to 2.54 during the time from 2010 to 2016 (47), it still significantly outnumbered by US and countries in Europe (48). It has been reported that the nurse and midwife ratio per 1,000 population is approximately 12.66 in Australia and 8.33 in UK in 2016 and approximately 8.55 in US in 2015 (49). Prior research has documented that the likelihood of burnout was increased by 23% due to each additional patient a nurse must attend to (33). Meanwhile, the frequent occurrence of workplace violence against medical workers in recent years in Chinese hospitals, which has been attributed to various causes including lack of adequate primary health-care system and effective physician-patient communication (50), has also been linked to higher incidences of burnout among Chinese nurses (51). As mentioned above, nurses in China have been exposed to excessive workload and requirements combined with inadequate staffing and limited resources; this increases the likelihood of nurses' appraising their situation as stressful, which might in turn contribute to higher levels of burnout among Chinese nurses.

Our study also extends the existing literature by examining sleep quality as a mediator in the relationship between perceived stress and burnout among nurses. Prior studies showed that poor sleep quality (52, 53) or inadequate sleep (54) and perceived stress (55) were risk factors for burnout among medical student population. Perceived pressure and sleep were related to burnout among faculty at doctoral research universities (56). A recent cross-sectional study conducted among osteopathic medical students also found that higher perceived stress and poorer sleep quality were associated with all three dimensions of burnout (57). Results of our study reveals that perceived stress might not only exert a direct effect on burnout, but could also have indirect effect on burnout through sleep quality, indicating that nurses with higher levels of perceived stress tend to report poorer sleep quality and there is an increased risk on the development of burnout. This finding confirms previous studies revealing the positive correlation between perceived stress and poor sleep quality (58, 59). A possible explanation might be that sleep has been revealed to play a vital role in restoring daily functioning and regulating emotional experiences, which could mediate the relationship between stress and negative effects of burnout and help the brain process emotionally stressful events in adaptive ways (60). Sleep quality influences how individuals react to stressful events, for there is evidence showing the link between sleep deprivation and increased sensitivity to stressful stimuli and events (60) and lack of sufficient healthy sleep may result in the increase of negative emotional reactivity and reduce positive reactions to positive events (61). Inversely, one's response to daytime stressful events involves the ability to disengage from active wake processing, which could prevent the normal sleep process from being initiated (60). This could explain the bidirectionality of poor sleep quality and the perception of stress.

Meanwhile, the present study lends support to prior research conducted among nurses (62) and physicians (63, 64) confirming the finding that poor sleep quality was positively correlated with burnout. This might be due to the reason that burnout, based on the Conservation of Resources (COR) Theory (65), results from a continuous loss of resources that individuals lack the opportunities to replenish, whereas sleep can serve to halt the spiral of resource loss and contribute to the obtainment of other resources (such as good performance) (64). The dysregulation of hypothalamic–pituitary–adrenal (HPA) axis, which is commonly observed both among individuals reporting burnout and among those with sleep disturbances, might partially explain the relationship existing between sleep quality and burnout (64). It has been suggested that poor sleep quality is associated with a hyperactive state which is also integral in burnout and may cause an increased activation of the HPA axis, giving rise to a prolonged increase in the allostatic load (66). An increased activation of the HPA axis, which is the central stress response system and responsible for one's long-term adaptation to stress, could play a mediating role in the relationship between burnout and sleep disturbance (67). Another explanation might be that impaired sleep is closely related to sustained cognitive activation, or the inability to unwind or disengage from thoughts of work during leisure time, which was also found to predict burnout (68). An incomplete recovery pathway was shown to link from work demands to impaired sleep and, in the long run, to the development of burnout (68).

## Limitations

This study is subject to several limitations despite the above-mentioned strengths. First, the cross-sectional design of this study provides no evidence for the existence of causal relationships between perceived stress, sleep quality and burnout. The associations among stress, sleep and burnout might be bidirectional. Whereas, it is plausible that perceived stress exerted effects on burnout both directly and indirectly through the mediating role of sleep quality, it might be just as likely that burnout leads to perceived stress mediated by poor sleep quality. Further prospective studies are therefore necessary to clarify the directions of the associations among perceived stress, sleep quality and burnout. Second, the potential response bias of the self-reported measures used in this study may lead to an underestimation or overestimation of the associations between the variables. Third, all nurses participating in this study were recruited from public tertiary hospitals in Liaoning Province in Northeast China. The generalizability of the study results to a broader nursing population requires confirmation by further research.

## Conclusions

This study represents the first attempt to explore sleep quality as a mediator in the relationship between perceived stress and job burnout among Chinese nurses. Our finding reveals that both perceived stress and poor sleep quality exhibited strong positive associations with burnout among Chinese nurses and perceived stress might exert effects on burnout both directly and indirectly through the mediating role of sleep quality. It could therefore be implied that efforts to reduce burnout among nurses might be expected to benefit from interventions for coping with perceived stress rather than the less readily modifiable work-related factors. Also, it is advisable that resources to promote healthy sleep practices should be provided at the organizational level (63) and more importance should be attached to sleep health for reducing burnout among the nursing staff.

## Data Availability Statement

The raw data supporting the conclusions of this article will be made available by the authors, without undue reservation.

## Ethics Statement

The studies involving human participants were reviewed and approved by the Committee on Human Experimentation of China Medical University. The participants provided their written informed consent to participate in this study.

## Author Contributions

XY conceptualized, designed and supervised the project. YS reviewed the literature and wrote the manuscript. FY collected, analyzed, and interpreted the data. KS provided assistance and revised the manuscript. All authors read and approved the final manuscript.

## Conflict of Interest

The authors declare that the research was conducted in the absence of any commercial or financial relationships that could be construed as a potential conflict of interest.
